# Properties of Neurons Derived from Induced Pluripotent Stem Cells of Gaucher Disease Type 2 Patient Fibroblasts: Potential Role in Neuropathology

**DOI:** 10.1371/journal.pone.0118771

**Published:** 2015-03-30

**Authors:** Ying Sun, Jane Florer, Christopher N. Mayhew, Zhanfeng Jia, Zhiying Zhao, Kui Xu, Huimin Ran, Benjamin Liou, Wujuan Zhang, Kenneth D. R. Setchell, Jianguo Gu, Gregory A. Grabowski

**Affiliations:** 1 Division of Human Genetics, Cincinnati Children’s Hospital Research Foundation, University of Cincinnati College of Medicine, Cincinnati, Ohio, United States of America; 2 Division of Developmental Biology, Cincinnati Children’s Hospital Research Foundation, University of Cincinnati College of Medicine, Cincinnati, Ohio, United States of America; 3 Division of Pathology and Laboratory Medicine, Cincinnati Children’s Hospital Research Foundation, University of Cincinnati College of Medicine, Cincinnati, Ohio, United States of America; 4 Department of Pediatrics, University of Cincinnati College of Medicine, Cincinnati, Ohio, United States of America; 5 Department of Anesthesiology, University of Cincinnati College of Medicine, Cincinnati, Ohio, United States of America; 6 Department of Pharmacology, Hebei Medical University, Shijiazhuang, China; 7 Synageva BioPharma Corp., Lexington, Massachusetts, United States of America; University of Cincinnati, UNITED STATES

## Abstract

Gaucher disease (GD) is caused by insufficient activity of acid β-glucosidase (GCase) resulting from mutations in *GBA1*. To understand the pathogenesis of the neuronopathic GD, induced pluripotent stem cells (iPSCs) were generated from fibroblasts isolated from three GD type 2 (GD2) and 2 unaffected (normal and GD carrier) individuals. The iPSCs were converted to neural precursor cells (NPCs) which were further differentiated into neurons. Parental GD2 fibroblasts as well as iPSCs, NPCs, and neurons had similar degrees of GCase deficiency. Lipid analyses showed increases of glucosylsphingosine and glucosylceramide in the GD2 cells. In addition, GD2 neurons showed increased α-synuclein protein compared to control neurons. Whole cell patch-clamping of the GD2 and control iPSCs-derived neurons demonstrated excitation characteristics of neurons, but intriguingly, those from GD2 exhibited consistently less negative resting membrane potentials with various degree of reduction in action potential amplitudes, sodium and potassium currents. Culture of control neurons in the presence of the GCase inhibitor (conduritol B epoxide) recapitulated these findings, providing a functional link between decreased GCase activity in GD and abnormal neuronal electrophysiological properties. To our knowledge, this study is first to report abnormal electrophysiological properties in GD2 iPSC-derived neurons that may underlie the neuropathic phenotype in Gaucher disease.

## Introduction

Gaucher disease (GD) is an autosomal recessive lysosomal storage disease caused by accumulation of glucosylceramide and glucosylsphingosine [1] as a result of defective acid β-glucosidase (GCase, EC3.2.1.45) activity. Over 350 mutations have been identified at *GBA1* resulting in heterogeneous disease phenotypes [2]. Clinical manifestations of GD have been classified into three types. Type 1 (GD1) is a non-neuronopathic form that accounts for 90% of GD cases in the Western world. The signs of GD1 include hepatomegaly, splenomegaly, bone pain and fractures, anemia and thrombocytopenia [1]. Patients with GD1 can survive into adulthood. GD1 variants do not manifest early onset of progressive primary CNS disease. GD type 2 (GD2) is an acute neuronopathic disease with onset in the first months and progression to death between 3 and 24 months. In addition to visceral involvement, GD2 patients have progressive CNS disease that includes bulbar signs, ataxia, and seizures [1,3]. GD type 3 (GD3) has variable signs of chronic progressive neuronopathic and visceral involvement. Such patients can survive into the 2^nd^ to 5^th^ decades [1].

Two available treatment approaches for the visceral manifestations of GD include enzyme supplementation, a.k.a. enzyme replacement therapy (ERT), and inhibition of substrate production or substrate reduction therapy (SRT) [4,5]. ERT safely improves the liver, spleen, and bone marrow and hematological disease, but because the enzyme does not penetrate the blood-brain barrier in therapeutically effective amounts, the CNS remains untreated. Small molecules that inhibit glucosylceramide synthase, i.e., SRT, may penetrate into the brain and inhibit glucosylceramide synthase to alter glucosylceramide levels, but they have not shown effectiveness in correction of the neurologic phenotype [6]. Development of effective therapy for patients with the neuronopathic GD variants and other neurodegenerative diseases is hindered by a poor understanding of their pathologic mechanisms. Accumulation of glucosylceramide and glucosylsphingosine in patient brains and visceral tissues has been well documented [7,8]. Gaucher cells, the engorged tissue macrophages of GD patients, may generate excess cytokine and proinflammation in GD organs, including brain [9–11]. Glucosylceramide accumulation, greatly elevated glucosylsphingosine, and neuron loss are prominent features in the brain of neuronopathic variants [12–17]. The association of increased substrate(s) levels and severity of neuronopathic GD implicates the accumulated substrates as being directly involved in the CNS disease progression [18].

In addition to substrate accumulation, protein aggregation, e.g. α-synuclein (αSYN) and amyloid precursor protein (APP), is found in GD mouse brains [19–21]. That mutant GCase proteins potentiate αSYN aggregation is supported by the fact that the αSYN accumulation in GD mouse brains can be corrected by CNS expression of human GCase [22]. Such studies have explored the underlying mechanisms of mutations in *GBA1* as important genetic modifiers for Parkinson and Lewy Body disease [21,23–25]. The GCase dysfunction and substrate accumulation in the brains of GD mice may cause defects in lysosome and autophagy function, which result in toxic protein (αSYN and APP) aggregation in the cells. The protein aggregates colocalize with mitochondria and affect mitochondrial function [21,26,27]. Reduced mitochondrial membrane potential and decreased ATP production were determined in the late stage of neuronopathic GD mouse models [21,26]. Thus, defective GCase function and glucosylceramide and glucosylsphingosine accumulation are anticipated to be the risk factors for mitochondrial dysfunction and the multi-proteinopathies in neuronopathic GD [21]. Whether the glucosylceramide and glucosylsphingosine accumulation could affect neuron function remains to be determined.

The generation of induced pluripotent stem cells (iPSCs) provides the opportunity to develop human disease cell models that would otherwise be inaccessible [28,29]. The reprogramming of somatic human cells to iPSCs, and their subsequent differentiation to disease-relevant cells allows the investigation of cell type specific disease mechanisms and the potential for autologous cell replacement therapies for human diseases. Human iPSCs have been derived from GD1 heterozygotes (RecNcil/wt; L444P/wt and N370S/wt), GD1 homozygotes (N370S/N370S), GD2 (L444P/RecNciI, L444P/G202R), and GD3 (L444P/L444P) fibroblasts [30–33]. These iPSCs have been differentiated into macrophages and neurons, which show defective GCase activity that can be rescued with pharmaceutical chaperones [30–32]. However, the neuronal electrophysiology has not been determined in iPSC derived cells from those studies, which will be important for understanding the pathological mechanism of neuronopathic GD.

Here, human fibroblasts from three GD2 patients (L444P/P415R, G325R/C342G, and one homozygous for alleles with two variants with genotype L444P;E326K/L444P;E326K) and two non-disease controls (one with no *GBA1* pathological variants and a heterozygote for L444P plus benign 1483G>C and 1497G>C substitutions on one allele) [34–39] were reprogrammed to iPSCs, and further differentiated to neural precursor cells (NPCs) and neurons. These cells were evaluated for their cell type specificity and biochemical and electrophysiological properties as a cell model for neuronopathic GD.

## Materials and Methods

### Materials

Fibroblasts derived from three patients with GD2 (GM01260, GM02627, GM08760) and one individual heterozygous carrier (GM00878) were from the Coriell Institute for Medical Research (S1 Table) [34–39]. Normal human foreskin fibroblasts (HFF10), a generous gift from Dr. Susanne Wells, were from a non-disease donor (control) with a wild-type *GBA1* DNA sequence (S1 Fig.) [40]. Human embryonic stem cells (hESCs) (H9) were from the WiCell Research Institute (Madison, WI) [41]. The following were from commercial sources: hESC-qualified matrigel (BD Biosciences, San Jose, CA), N2 supplement, DMEM supplemented with 10% fetal bovine serum, and 1x non-essential amino acids (Invitrogen, Grand Island, NY), mTeSR1 (StemCell Technologies, Vancouver, Canada).

### Generation of iPSC lines from control and GD2 fibroblasts

Human control and Gaucher disease fibroblasts were cultured in Mouse Embryonic Fibroblasts (MEF) media consisting of DMEM supplemented with 10% fetal bovine serum and 1x non-essential amino acids. *GBA1* coding region in the control and mutant cells were DNA sequenced. For iPSC generation, fibroblasts were either transduced with recombinant VSV-G pseudotyped polycistronic lentiviral particles co-expressing reprogramming factors Oct4, Klf4, Sox2, cMyc and dTomato [42] or nucleofected (program U20) with episomal plasmids pCLXE-hOct3/4-shp53, pCLXE- hSox2-Klf4, pCLXE- hLmyc-Lin28, and pCLXE-GFP [43]. For lentiviral iPSC generation, fibroblasts were plated on hESC-qualified matrigel and 3–5 days post transduction, MEF media was replaced with mTeSR1, and cultures were subsequently fed daily with mTeSR1. After ∼3 weeks putative iPSC colonies were identified and exposed to dispase for 5 minute. Discrete colonies were manually excised and replated in mTeSR1 on matrigel-coated dishes. The derivation and quality testing of the control iPSC line derived from a healthy individual that was used in this study has been described [40]. For episomal plasmid iPSC generation, six days post-nucleofection, fibroblasts were replated in MEF media in a gelatin-coated 10 cm dish containing 1.07 x 10^6^ irradiated mouse embryonic fibroblasts (MEFs). Starting on day 7 post-transfection, cells were fed daily with DMEM/F12 media supplemented with 20% knockout serum replacement, 1 mM L-glutamine, 0.1 mM β-mercaptoethanol, 0.1 mM non-essential amino acids, and 4 ng/mL basic FGF (all from Invitrogen). Approximately 2 weeks later, discrete colonies with hESC-like morphology were manually excised and replated in mTeSR1 media (Stem Cell Technologies) in tissue culture dishes coated with hESC-qualified matrigel (Becton Dickinson). A number of iPSC lines derived from each donor with stable and typical hESC-like morphology were then expanded independently in mTeSR1 on matrigel-coated dishes. For passaging, iPSCs were exposed to dispase for 5 minutes, washed, and gently triturated before replating. iPSC lines were cryopreserved in mFreSR (StemCell Technologies). All cultures were maintained in a 5% CO2/air environment. All the experiments with iPSCs in this study were approved by institutional Embryonic Stem Cell Research Oversight (ESCRO) at Cincinnati Children’s Hospital Medical Center.

### Neural induction of iPSCs

Populations of self-renewing neural precursor cells (NPCs) were obtained using the commercially-available embryoid body-based directed differentiation method (Stem Cell Technologies). In brief, neural aggregates were formed by culturing iPSCs in AggreWell800 plates in STEMdiff Neural Induction Medium. After 5 days of culture, the aggregates were plated onto poly-L-ornithine/laminin (PLO/L) coated tissue culture plates where they attached and formed rosettes. NPCs derived from the rosette structures were maintained on PLO/L plates in NPC medium (DMEM/F12 with 0.5x N2 supplement, Invitrogen), 0.5x B27 supplement (Miltenyi) and 10 ng/mL bFGF (Invitrogen). Cells were passaged using Accutase (Innovative Cell Tech) per manufacturer’s instructions and replated at 100,000 cells/cm^2^ for maintenance and at 20,000 cells/cm^2^ for differentiation. For dopaminergic patterning, NPC medium was replaced with Neurobasal media containing 1x N2 supplement, 2 mM L-glutamine, 50 μg/mL ascorbate, 100 ng/mL SHH, and 100 ng/mL FGF8 (neuron differentiation medium) [44]. For CBE (conduritol B epoxide, a specific covalent inhibitor of GCase) treatment of control cells, CBE (2 mM) was added to the medium during the last 7 days through 14 day neuron differentiation.

### Immunofluorescence staining

The NPCs and neurons grown on cover slips were fixed in 4% paraformaldehyde in 1x PBS for 1 hr at 4°C and washed twice with 1x PBS. The fixed cells were incubated in 0.3% Triton X 100 for 30 min to 1 hr, and treated with 50 mM NH_4_Cl in 1x PBS for 15 min followed by a 1x PBS wash. The cells were blocked for 1 hr at RT in blocking buffer (5% goat serum and 0.4% Triton X 100 in PBS). Primary antibodies diluted in the Blocking buffer were applied to the cells and incubated overnight at 4°C. After PBS washing (3x 10 min), the secondary antibodies, goat anti-mouse or/and goat anti rabbit conjugated with Alexa Fluor 488 or Alexa Fluor 633 (1: 1500) in blocking buffer were applied and incubated for 1 hr at RT. Following 1x PBS washes (3x 10 min), the cells were mounted with VECTASHIELD mounting medium containing DAPI (Vector H1200). The signals were observed under Zeiss Apotome fluorescence microscope. The antibodies used: rabbit anti-nestin (1:125; Millipore, AB5922), rabbit anti-sox2 (1: 100; Millipore, AB5603), rabbit anti-Map2 (1:200; Millipore, AB5622), mouse anti-α-synuclein (1:10; Invitrogen, 18-0215), mouse anti-synaptophysin (1:100; Millipore, MAB5258), rabbit anti-Tyrosine hydroxylase (1:100; Chemicon, AB152), mouse anti-Tuj1 (1:100; Millipore, MAB1637), mouse anti-Lamp1 conjugated with Cy5 (1:100; Abcam, ab25222), and rabbit anti-human GCase (1:100) [45].

### GCase activity analyses

The cells were homogenized in 1% sodium taurocholate/1% Triton X-100, and GCase activities were determined fluorometrically with 4MU-Glc in 0.25% Na taurocholate and 0.25% Triton X-100 [46]. Protein concentrations of cell lysates were determined by BCA assay using BSA as standard.

### Immunoblotting

The cells were homogenized in M-PER and the extracts were subjected to electrophoresis on NuPAGE 4–12% Bis-Tris gel. The proteins were transferred to Hybond-ECL nitrocellulose membranes as described [47]. Human GCase proteins in cell lysate were detected on immunoblots with rabbit anti-human GCase antibody (1/1000). α-Synuclein in cell lysate was detected using monoclonal anti α-Synuclein antibody (1:60; Invitrogen, 18-0215). β-actin in the same blot was detected by monoclonal anti- β-actin antibody. The signals were detected with ECL detection reagent (GE Healthcare) according to manufacturer’s instructions.

### Glucosylceramide and glucosylsphingosine analyses

Glycosphingolipids in the cells were extracted as described with modification [48]. Cell pellets were lysed in 700 μL of ddH2O with trituration and vortexing. Fraction (630 μL) of this suspension was dried under N2, then resuspended in 0.6 mL of 0.3N KOH in methanol and incubated for 1 hr at 37°C. The remainder was used to measure protein concentrations. Phenolphthalein (20 μL, 1 mg/mL in ethanol) was added as pH indicator. The solutions were neutralized with 3N HCl (∼45 μL). The lipids were extracted by addition of chloroform (300 μL) and water (50 μL) to bring the fraction of each solvent to methanol/chloroform/water (2:1:0.33, v/v/v), and shaken for 15 min at room temperature. To remove non-lipids contaminants, the samples were brought to 5 mL and desalted on the Sephadex G-25 as described [49]. Quantifications of glucosylceramides and glucosylsphingosine were carried out by LC-ESI-MS/MS as described [48]. Glucosylceramide and glucosylsphingosine levels were normalized to the lysate protein concentration.

### Electrophysiology

Coverslips with iPSC-derived neurons (cultured 14 days in neuron differentiation medium) from three GD2 patients (GM01260, GM02627, GM08760), one control and control with CBE were placed in a 0.5-ml microchamber, perfused by the normal bath solution at the speed of 2 ml/min. The normal bath solution contained (in mM) 145 NaCl, 5 KCl, 2 MgCl_2_, 2 CaCl_2_, 10 Glucose, and 10 HEPES (pH adjusted to 7.3 with NaOH and osmolarity to 320 mOsm with Sucrose). Whole-cell patch-clamp recordings were performed from these cultured iPSC-derived neurons. The neurons were determined by neuronal appearance that has a compact cell body with 2–3 long projections resembling axons or dendrites in appearance. The types of neurons in the culture were not identified with live cell markers. Pipettes were made from borosilicate glass capillaries by the puller of P-97 (WPI instruments) and had resistances of 3–6 MΩ when filled with internal solution. The pipette internal solution contained (in mM) 135 K-Gluconate, 5 KCl, 2.4 MgCl_2_, 0.5 CaCl_2_, 5 EGTA, 10.0 HEPES, 5.0 Na_2_ATP, 0.33 GTP-Tris salt. pH was adjusted to 7.35 with KOH and osmolarity was adjusted with sucrose to 320 mOsm. Electrophysiological signals were recorded using an Axon 200B amplifier, filtered at 2 KHz, and sampled at 5 KHz with the pClamp 9.0 software (Molecular Device). To test membrane excitability and action potential firing, cells were recorded under current-clamp configuration. Current steps were applied to cells from −100 pA to 200 pA with each step at 20 pA. The step currents were injected at the duration of 1 s for each step and the interval between each step was 3 s. To test voltage-activated currents, cells were held at −70 mV and voltage steps were applied to the cells from −90 to +40 mV with each step at 10 mV. The duration of the voltage steps was 250 ms and applied at the interval of 500 ms.

### Karyotype and pluripotency analyses of iPSCs

Standard G-banded karyotype analyses of control and GD2 derived iPSC lines were performed by the Cytogenetics Laboratory at Cincinnati Children’s Hospital Medical Center. At least 20 metaphases were analyzed per iPSC line and clones in which the karyotypes were normal were used for further experiments. Analysis of *in vivo* differentiation capacity was assessed with teratoma assays. Undifferentiated iPSCs were injected subcutaneously into immune compromised NOD/SCID GAMMA C-/- mice. Teratomas formed within 6–12 weeks. Teratomas were excised, fixed, embedded in paraffin, sectioned and stained with hematoxylin and eosin for histological examination as described [40].

### Immunofluorescence staining

For analysis of pluripotency marker expression, iPSC cultures were fixed for 10 min at room temperature with 3.7% paraformaldehyde in PBS. Cells were then permeabilized for 10 min with PBS containing 0.5% Triton X-100 and incubated for 30 min at room temperature in blocking buffer (10% normal donkey serum in PBS). Antibodies to human Oct4 (Santa Cruz, sc-5279) and Nanog (Abcam, ab21624) were diluted in blocking buffer at 1:500 and incubated with cells overnight at 4°C. After incubation with fluorescent-labeled secondary antibodies, cultures were visualized using fluorescent microscopy. 4′,6-diamidino-2-phenylindole (DAPI) was used for nuclear counterstaining.

### Deglycosylation

Cell pellets were homogenized in M-PER (Mammalian Protein Extraction Reagent, Thermo, cat#78501) in the presence of protease inhibitor cocktail (Sigma, Cat#P8340). Protein concentration was determined by the BCA assay. For N-Glycanase (PROzyme, GKE-5006) reactions, proteins in the lysates were denatured at 100°C for 5 min in 0.1% SDS and 50mM β-mercaptoethanol. After cooling, NP-40 was added to 0.75% followed by N-Glycanase (0.1U/mg protein) and incubated for 24 hrs at 37°C. For Endo H (New England BioLabs, P0702S) digestion, the lysate was heated at 100°C for 10 min in glycoprotein denature buffer (0.5% SDS and 0.04M DTT). The cooled samples was incubated with Endo H (25 U/μg protein) in G5-reaction buffer (0.05M Sodium Citrate, pH5.5) for 24 hrs at 37°C. The proteins were resolve on 10% gel and immunoblotted with rabbit anti-human GCase antibody as described above.

### Statistical analyses

The data were analyzed by Student's t-test or ANOVA test using GraphPad Prism 5.

## Results

### Generation of human GD2 iPSC and differentiation to neural progenitor cells (NPC) and neurons

Primary human fibroblasts from three patients with GD2 [GD2-1260 (GM01260), GD2-2627 (GM02627) and GD2-8760 (GM08760)] and two controls [normal (HFF10) and heterozygous carrier (GM00878)] with typical fibroblast morphology were reprogrammed into iPSCs using a polycistronic lentiviral vector or episomal plasmids expressing Oct4, Sox2, Klf4, and cMyc (Table 1) as described [42,43]. The reported mutations in *GBA1* in the fibroblasts or iPSCs and the normal GBA1 in control cells were confirmed by DNA sequencing (Supporting Information S1 Fig.). An additional mutation was identified in GD2-8760 in this study (Table 1). The pluripotency of iPSCs were determined following standard protocol as described [40] demonstrating successful reprogramming of control and GD2 fibroblasts to pluripotent stem cells and pluripotency *in vivo* (S2 Fig.). All the iPSC clones used had normal karyotypes (S3 Fig.). At least two clones of iPSC derived from three lines of GD2, 1 control and 1 carrier were differentiated to NPCs and neurons for the subsequent analyses. Lysosomal localization of GCase in stem cells was determined by immunofluorescence in normal control and GD2-1260 iPSCs. Human GCase protein was detectible in control iPSCs and human ES cells, and colocalized with Lamp 1, a lysosomal marker, indicating lysosomal localization of GCase. GCase in GD2-1260 iPSCs was barely detectible in the cytoplasm and did not show colocalization with Lamp 1, indicating GCase deficiency in GD2 iPSCs (S2 Fig. C).

**Table 1:** Fibroblast lines from Gaucher disease patients and control human used for iPSC reprogramming and neural differentiation.

ID	Diagnosis	Genotype	# of iPSC clones	Reprogramming method
GM01260 (GD2-1260)	TYPE 2	L444P /P415R	12	Lentivirus
GM02627 (GD2-2627)	TYPE 2	G325R / C342G	6	Episomal
GM08760 (GD2-8760)	TYPE 2	L444P:E326K/ L444P:E326K	6	Episomal
GM00878 (Carrier)	Heterozygote	carrier for L444P and 1483G>C and 1497G>C	6	Episomal
HFF10 (Control)	Normal human	No *GBA1* mutation	3	Lentivirus

**Fig 1:**
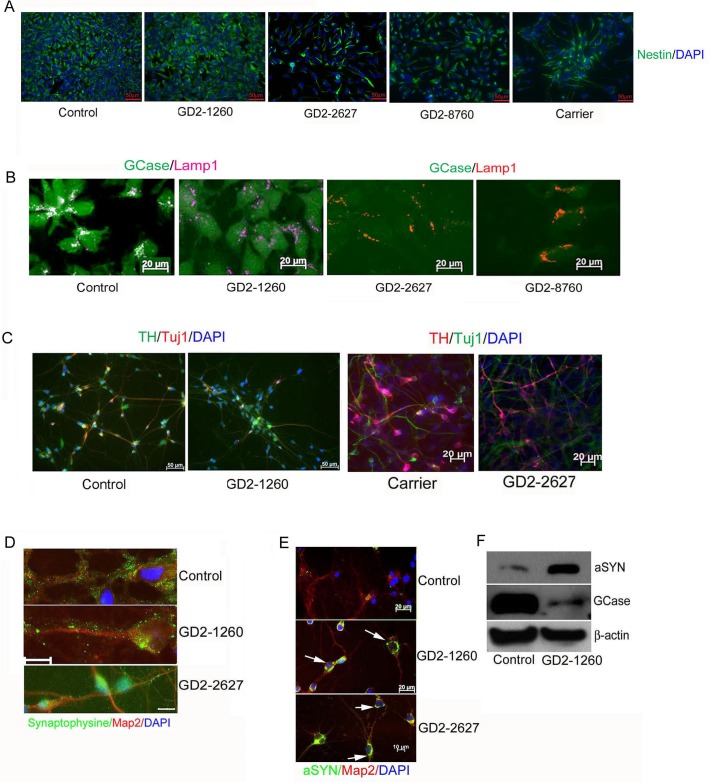
Characterization of iPSC-derived NPCs and neurons. (A) Neural stem cell marker staining. NPCs showed positive immunofluorescence signals for Nestin (green). Scale bars are 50 μm. (B) GCase expression and localization. GCase (green) in control NPCs colocalized with lysosomal marker Lamp1 (violet or red). Dual GCase and Lamp1 signal in control cells shows white color. GD2-1260, GD2-2627 and GD2-8760 NPCs had Lamp1 signal and low level GCase. Scale bars are 20 μm. (C) Tuj1 positive neurons and tyrosine hydroxylase (TH) positive DA neurons detected in control, GD heterozygous carrier and GD2 iPSC-derived neurons at 14 days in neuron differentiation medium. (D) Synaptic protein formed in differentiated neurons. The NPCs in neuron differentiation medium for 14 days were stained positive by anti Map2 antibody (red). These iPSC-derived neurons of control, GD2-1260 and GD2-2627 were positive for presynaptic protein, synaptophysin (green). Scale bar is 10 μm for 3 images. (E) Alpha-synuclein (aSYN) aggregates (green, arrow) detected in GD2-1260 and GD2-2627 neurons cultured for 30 days. (F) Immunoblot. aSYN level (14 kD) was increased and GCase level was low in GD2-1260 neurons cultured 14 days. β-actin is the loading control. DAPI stains nuclei.

The generation of NPCs from iPSCs was accomplished using an embryoid body method in which uniform cell aggregates are formed in Aggrewell dishes with a defined media for neural induction [50]. The resulting neural aggregates were plated on poly-L-ornithine/laminin (PLO/L) coated tissue culture plates and within a day, rosette structures formed NPCs that grew out of the isolated rosettes were self-renewing and expressed signals for the neural stem cell markers, nestin and sox2 (Fig. 1A and S4 Fig. A). Lysosomal localization of human GCase in control NPCs was determined by colocalization with Lamp 1. Low level signals for GCase expression in GD2-derived NPCs (GD2-1260, GD2-2627 and GD2-8760) did not show colocalization with Lamp 1 (Fig. 1B).

NPCs were grown on PLO/L plates in defined media containing FGF8 and SHH following dopaminergic (DA) neuron differentiation protocol [44]. In this differentiation condition, neuronal markers Map2, Tuj1, and NeuN, as well as DA specific TH were detected in all three GD2 and control cultures at about 2 weeks (Fig. 1C and S4 Fig. B). The percent of cells that expressed TH varied from around 10 to 25%. Neurons could be maintained in this media for at least 30 days. To demonstrate the maturation of the neurons, cells in 30 day neuron cultures were shown to express synaptophysin (Fig. 1D), a presynaptic protein, suggesting the formation of synapses on the neuronal processes. Consistent with our previous report, in these 30 day neuron cultures, αSYN aggregates were detected in GD2 neurons by immunofluorescence, whereas control neurons showed low-level αSYN staining only after enhancing the contrast (Fig. 1E) [19]. Immunoblots of proteins extracted from 14 day neuron cultures showed more 14 kD αSYN (6.4-fold) and less GCase in GD2 neurons compared to the control (Fig. 1F). High molecular weight species of αSYN was not detected in these cells.

### Biochemical characterizations of GD2 cells

**Fig 2:**
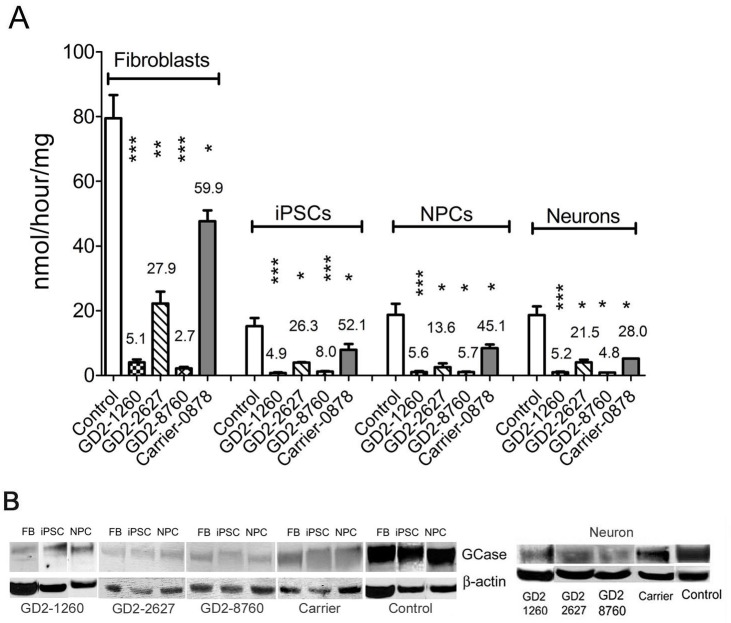
GCase deficiency in iPSC derived cells. (A) GCase activity. In the control cells, the GCase activity was lower in the derived cells than in the fibroblasts: iPSCs (20%), NPCs (27%) and neurons (19%) of the activity in the fibroblasts. GCase activity in GD2-1260 and GD2-8786 was <5% in fibroblasts, iPSCs and NPCs, and 6–12% in derived neurons compared to the control cells. GCase activity in GD2-2627 cells was 11–28% of control level. Carrier cells had GCase activity >40% of control level in each cell type. Student’s t test (*, p<0.05; **, p<0.01; ***, p<0.0001). (B) GCase protein. GCases were detected by anti-human GCase antibody. GCase protein band was ∼55 kDa in all GD2 and control cell types. GD2 cells had low levels of GCase protein than that in the carrier and control cells. 100 μg protein lysate was loaded on each lane and β-actin is the loading control.

GCase activity was measured in lysates of control and GD2 fibroblasts, iPSCs, NPCs, and neurons. The GCase activity levels varied with cell type consistently over each genotype. In the control cells, GCase activity in iPSCs was 19% of the activity from fibroblasts, NPCs were 24% and neurons were 23% (Fig. 2A). GCase activities in the GD2 and heterozygous (Carrier) cell lines were consistently lower than those in control cells in all 4 cell types (fibroblasts, iPSCs, NPCs and neurons). GCase activities were about 4.9–5.6% of control level in GD2-1260 cells, 13.6–27.9% in GD2-2627, and 2.7 to 8.0% in GD2-8760 and the heterozygous (Carrier) had GCase activity at about 28–60% of control levels across fibroblasts, iPSCs, NPCs and neurons (Fig. 2). GCase protein from GD2 patients and control had apparent similar molecular weights in fibroblasts, iPSCs, NPCs and neurons (Fig. 2B). Consistent with previous reports on fibroblasts [34,35,51], low levels of GCase proteins were determined in GD2 iPSCs, NPCs and neurons. The presence of complex oligosaccharides on GCase was similar in each cell type as determined by Endo-H and N-glycanase digestion (S5 Fig.). These results demonstrate the deficiency of GCase activities and proteins in GD2 fibroblast-derived iPSCs, NPCs and neurons, and confirm the retention of abnormal GCase activity in neurons derived from GD2 iPSCs.

**Fig 3:**
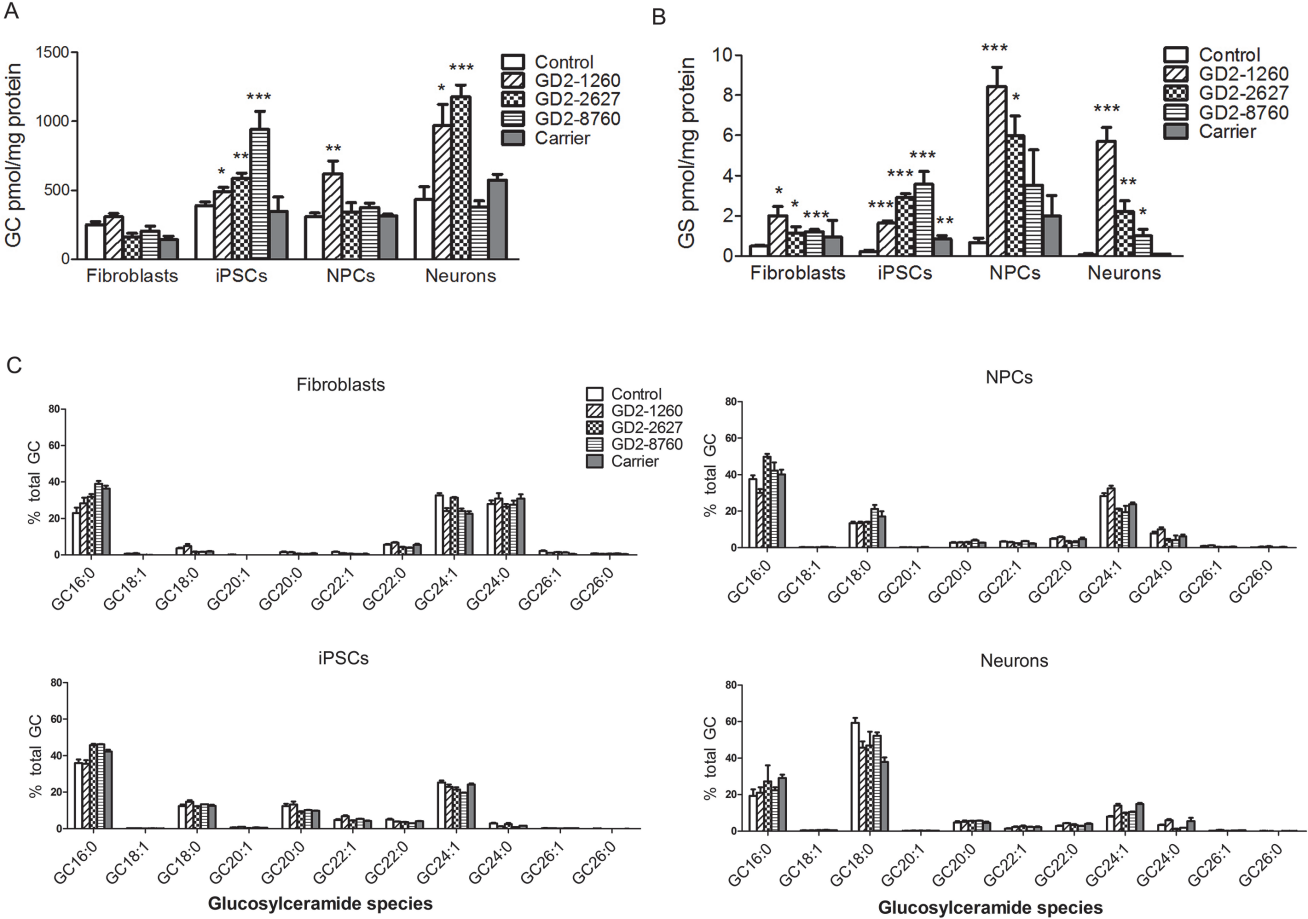
Glycosphingolipids analyses of substrate accumulation by LC/MS. (A) Glucosylceramide (GC) was increased in GD2-8760 iPSCs, GD2-1260 NPCs and neurons and GD2-2627 neurons (14 d) compared to controls. There was no significant difference in glucosylceramide levels between GD2 and control fibroblasts. (B) Glucosylsphingosine (GS) levels were increased in GD2 fibroblasts, iPSCs, NPCs and neurons (14 d) compared to control cells. Glucosylceramide and glucosylsphingosine levels in fibroblasts, iPSCs and NPCs were normalized by protein level in cell lysate. Student’s t test (*, p<0.05; **, p<0.01; ***, p<0.0001). Data are Mean±S.E. (n = 3–10). (C) Glucosylceramide species. Proportion of each species analyzed by LC/MS was quantified and compared to total glucosylceramides (n = 3–12). Profiles of glucosylceramide species were distinct in each cell type. The profile of the accumulated glucosylceramides in GD2 cells corresponded to the species found in each of the control cell types.

Lipid analyses in the different cell types for total glucosylceramide (Fig. 3A) and glucosylsphingosine (Fig. 3B) concentrations showed some increases in glucosylceramide content and consistent increases in glucosylsphingosine content in GD2 *vs*. controls. Total glucosylceramide levels were significantly increased in all three GD2-iPSCs, GD2-1260 NPCs, and GD2-1260 and GD2-2627 neurons compared to the corresponding control cell types, but not elevated in GD2-2627 NPCs, GD2-8760 NPCs and GD2-8760 neurons (Fig. 3A). There was no difference in glucosylceramide levels between GD2 and control fibroblasts. Glucosylceramide levels in heterozygous (Carrier) were not different from the control in all cell types (Fig. 3A). The concentration of glucosylsphingosine was significantly increased in all three GD2 fibroblasts, GD2 iPSCs, and GD2 neurons. Glucosylsphingosine levels in GD2-1260 and GD2-2627 NPCs were significantly elevated. In GD2-8786 NPCs glucosylsphingosine levels were increased although they did not reach significance relative to controls (Fig. 3B). In heterozygous carrier cells, increased glucosylsphingosine was significant only in the iPSCs.

Glucosylceramide species profiles were distinct for each cell type (Fig. 3C). In the GD2 fibroblasts, iPSCs, and the iPSC-derived NPCs and neurons, the glucosylceramide profile corresponded to the species found in each of the control cell types. GC16:0, GC24:1 and GC24:0 lipids were predominant in fibroblasts, whereas GC16:0 and GC24:1 predominated in iPSCs and NPCs. The major glucosylceramide in neurons was GC18:0 in GD2 and control neurons, which is typical of brain glucosylceramide. Galactosylceramide was below the detection limit in all samples.

Collectively, these data indicate that GCase-deficiency results in abnormal lipid profiles in GD2 cells from distinct developmental stages that have not been reported previously.

### Electrophysiological properties of GD2 iPSC-derived neurons

Neuronal dysfunction in GD is strongly reinforced by the evidence of neurological involvement in GD2 patients [1,3] and the finding from the neuronopathic GD mouse model that had suppressed hippocampal long term potentiation [47]. To probe the basis for potential abnormal neuron function in GD2, we next investigated the electrophysiological properties of control and GD2 neurons by patch clamp recording methods. Three lines of iPSC-derived neurons from patients (GD2-1260, GD2-2627 and GD2-8760) and one line of control (HFF10) had been grown in neuron differentiation medium for 14 days at the time of recording. An unbiased electrophysiological approach was taken by selecting the cells with neuronal appearance that had a compact cell body with 2–3 long projections resembling axons or dendrites in appearance. The recordings were performed under the current-clamping configuration to determine membrane excitability (Table 2). The data were from two to three independent experiments each using one iPSC clone-derived neurons from each patient line and two iPSC clones-derived neurons from the normal control.

**Table 2:** Electrophysiological parameters of iPSC-derived neurons.

Genotype	Resting potential (mV)	Threshold (mV)	AP amplitude (mV)	Na current (at 0 mV, pA)	K current(at +40 mV, pA)
Control	-52,2 ± 2.2 (n = 25)	-26.6 ± 0.7 (n = 17)	97.9 ± 3.9 (n = 17)	-568.5 ± 80.0 (n = 21)	533.0 ± 59.0 (n = 29)
GD2-2627	-33.9 ± 2.1(n = 29)*	-30.3 ± 1.5 (n = 9)	54.5 ± 5.2 (n = 9)*	-253.9 ± 85.9 (n = 9)	344.5 ± 34.8 (n = 30)*
GD2-8760	-35.0 ± 1.6 (n = 36)*	-29.8 ± 1.7 (n = 13)	48.6 ± 7.6 (n = 13)*	-351.7 ± 82.1 (n = 16)	455.1 ± 37.8 (n = 36)
GD2-1260	-34.1 ± 2.4 (n = 14)*	-26.0 ± 1.5 (n = 4)	76.7 ± 7.5 (n = 7)	-490.9 ± 155.6 (n = 8)	628.6 ± 92.0 (n = 11)
CBE-GD	-31.7 ± 2.9 (n = 11)*	-26.8 ± 0.8 (n = 5)	75.0 ± 7.8 (n = 5)	-532.3 ± 95.9 (n = 8)	422.1 ± 45.6 (n = 15)

Mean with SEM; n, number of cells

*, significant difference between control and GD2 or CBE-GD cells determined by Anova.

**Fig 4:**
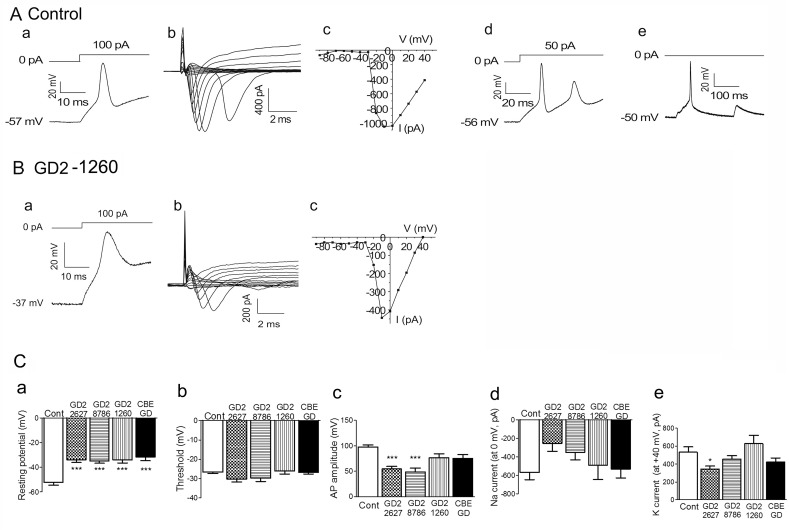
Electrophysiology of iPSC-derived neurons (14 d). (A) Action potential firing and voltage-gated sodium channels in control neurons. Control iPSC-derived neurons could fire a single action potential with membrane potential -57mV (a) or multiple action potentials (d) in response to current injection, or fire action potentials spontaneously (e). Voltage-gated sodium currents could be evoked by voltage steps (b) and the current-voltage relationship curve shows the activation of voltage-gated sodium channels (c). (B) Sample traces show membrane response to the injection of depolarizing current in GD2 iPSC-neurons. The resting membrane potentials of GD2-1260 iPSC-neurons were less negative (-37mV) although they were capable of firing action potentials (a). The voltage-gated sodium currents were smaller as shown in current trace (b) and the current-voltage relationship curve (c). (C) Action potential (AP) parameters of iPSC-neurons for control (Cont), GD2-1260, GD2-2627, GD2-8760 and CBE treated control iPSC-neurons (CBE-GD). (a) The resting membrane potential, (b) Threshold of action potentials, (c) The action potential (AP) amplitude, (d) Sodium current at the holding potential of 0 mV, (e) Potassium current at the holding potential of + 40 mV. Resting potential was significantly different between control and GD2 iPSC-neurons and CBE treated control iPSC-neurons. AP amplitudes of GD2-2627 and GD2-8760 iPSC-neurons and potassium current of GD2-2627 were reduced compared to the control. The data were from 2 to 3 independent experiments using one iPSC clone-derived neurons from each patient line (GD2-1260, GD2-2627, GD2-8760) and two iPSC clones-derived neurons from the control. Error bar represents the SEM. One-way ANOVA with Tukey's Multiple Comparison Test (* p < 0.05, ***p < 0.0001).

First, we determined whether iPSC-derived neurons are electrophysiological active. In 32 control iPSC neurons recorded, 17 (53%) fired action potentials in response to depolarizing current stimulations. In GD2 iPSC neurons recorded, a lower percentage of cells fired action potentials in response to depolarizing current stimulations: GD2-2627, 30% (9/30); GD2-8760, 36% (13/36); GD2-1260, 29% (4/14). The representative traces are shown in Fig. 4. These results indicated that many of the control and GD2 iPSC-derived cells exhibited functional properties of neurons. Some control iPSC neurons fired multiple action potentials in response to a single current stimulation (Fig. 4Ad) and some could spontaneously fire action potentials (Fig. 4Ae). In contrast, GD2 iPSC neurons neither fired multiple action potentials in response to a single current stimulation nor exhibited spontaneous action potential firing.

The inward currents were activated and inactivated rapidly, resembling voltage-gated sodium currents seen in native neurons [52]. Under the voltage-clamp configuration, voltage-activated inward currents could be evoked by voltage steps in control iPSC-derived neurons (n = 21/32) and GD2 iPSC-derived neurons: GD2-1260 (8/14), GD2-2627 (9/30) and GD2-8760 (16/36). Representative traces are shown in Fig. 4Ab&c and Fig. 4Bb&c. The current-voltage relationship curves for control iPSC-derived neurons (Fig. 4Ac) and GD2 iPSC-derived neurons (Fig. 4Bc) were consistent with the activation of voltage-gated sodium channels. These results suggest that action potential firing in control and GD2 iPSC-derived neurons were due to the activation of voltage-gated sodium channels expressed on these cells.

For control and GD2 iPSC-derived neurons that fired action potentials, the parameters of membrane potential and action potentials were compared (Fig. 4C and Table 2). The thresholds of action potentials for control and GD2 iPSC-derived neurons were not significantly different (Fig. 4Cb and Table 2). However, the resting membrane potentials (resting potential) of GD2 iPSC-derived neurons were significantly less negative compared to control neurons (Fig. 4Ca and Table 2). The action potential amplitudes (AP) are determined from the resting membrane potential to the peak of action potential. The AP amplitudes for GD2 iPSC-derived neurons, GD2-2627 and GD2-8760, were significantly different from control iPSC-derived neurons (Fig. 4Cc and Table 2). AP amplitude was reduced in GD2-1260, but did not reach statistical significance (Fig. 4Cc). Decreased AP amplitude could be caused by less negative resting membrane potential.

The alteration of membrane potential and AP amplitude suggests the disturbances in functional expression of sodium and potassium channels in GD2 neurons. The sodium and potassium currents were determined. Although GD2-2627, GD-1260 and GD2-8760 showed decreased sodium current, and GD2-2627 and GD2-8760 had reduced potassium current, respectively (Fig. 4Cd&e), only GD2-2627 reached significance on decreased potassium current compared to the control (Fig. 4Cd&e).

To confirm a functional link between defective GCase activity and electrophysiology in GD2 iPSC-derived neurons, the specific covalent inhibitor of GCase, CBE, was used. Control iPSC-derived neurons were treated with CBE for 7 days after neuronal differentiation. The CBE treated neurons had no detectable GCase activity and increased glucosylceramide and glucosylsphingosine levels (data not shown) confirming GCase inhibition. Compared to untreated controls, the CBE-treated iPSC-derived control neurons (CBE-GD) had significantly less negative resting potentials (Table 2 and Fig. 4Ca) that were comparable to those from GD2 iPSC-derived neurons. The AP amplitude and potassium current for CBE-GD neurons was reduced, but not significantly different from the control (Table 2 and Fig. 4C).

Taken together, these electrophysiological results indicate that control and GD2 iPSC-derived cells exhibited functional neuron properties. Interestingly, several electrophysiological parameters, resting membrane potential, AP amplitude, sodium and potassium currents, were altered in GD2 neurons. The similar electrophysiological properties in iPSC-derived cells from three GD2 patients and control neurons exposed to the GCase inhibitor CBE suggest that atypical neuronal function in these cells is linked to defective GCase, and contributes to the CNS phenotypes in GD.

## Discussion

iPSCs derived from GD patient fibroblasts have been used to obtain cultures of neurons and functional macrophages [19,30,31,33,53]. Differentiation of iPSC to neurons and DA neurons has been achieved [19,30,31], but the substrate profiles and neuron electrophysiology were not characterized in those studies. GD2 is an infantile, severe rapidly progressive neuronopathic disease [1,3,54]. To develop cell models for neuronopathic GD, iPSCs were derived from fibroblasts of three GD2 patients (Table 1) [34–39,51]. The iPSCs were differentiated into NPCs and neurons. The GD2 cells had decreased GCase activity and reduced GCase protein levels in cells analyzed at different neurodevelopmental stages that led to substrate accumulation, most significantly, glucosylsphingosine. Similar to the GCase deficient mouse models and previously reported GD iPSC-derived neuron, α-synuclein aggregates were detected in these iPSC-derived human GD2 neurons [19]. Functional analysis of the neurons demonstrated that both control and GD2 iPSC-derived neurons were capable firing action potentials. However, the GD2 iPSC-derived neurons showed abnormal membrane resting potential that was consistently detected in three independent GD2 iPSC lines carrying different mutations as well as in control iPSCs derived-neurons that were exposed to the GCase inhibitor CBE. These data indicate that GD2 iPSC-derived neurons harbor key disease relevant biochemical phenotypes and abnormal electrophysiological properties that may contribute neuronal pathologies in both developing and mature neural cell types in neuronopathic GD.

In GD2, neuronal loss and neurophagia occur in specific brain regions [1,54,55]. These changes and the storage inclusions that resemble twisted tubules structures are localized to apparently sensitive regions, including the oculomotor nuclei, hippocampus and cerebral cortex of GD2 brains [54,56]. The mechanistic connection for this underlying neuronal sensitivity is poorly understood [16,47,57]. Deficient GCase activity clearly disrupts substrate degradation and leads to elevated glucosylceramide and glucosylsphingosine in patient brains [12,15,17,57]. Glucosylsphingosine has been suggested to be the toxic agent leading to neuronal death, but this was assessed by feeding neuronal cultures with glucosylsphingosine in μmole amounts in the media [16]. Although glucosylsphingosine in surrounding media or interstitial spaces may contribute to the pathogenesis, this potential toxin is derived and accumulates intracellularlly due to defective glucosylceramide degradation which likely leads to increased glucosylsphingosine [58]. Here, glucosylsphingosine did accumulate in GD2 iPSC-derived cell types, particularly NPCs and neurons, with no apparent effect on survival of the cells. These findings as well as others [19,30,31] suggest a non-cell-autonomous nature of neuronal death in the CNS variants of GD. In comparison, fibroblasts from Sandhoff disease mice showed insufficient neural differentiation from iPSCs implying a cell autonomous role in the β-hexosaminidase deficiencies that affect GM2 ganglioside degradation in the lysosome [59].

Severe CNS involvement in GD2 patients include bulbar signs, ataxia, and seizures [1,3], which are postulated to be the result of defective GCase and subsequent neuronal dysfunction. Our findings of reduced long term potentiation in hippocampal tissue slices of neuronopathic GD mice [47] strongly suggest primary disturbed electrophysiological properties of the neurons in GD2 brains. iPSC-derived neurons allow the possibility of studying neuronal function in a more tractable setting compared to intact mice and primary neuron cultures. Here, GD2 iPSCs were differentiated into functional neurons with atypical electrophysiological properties including less negative resting membrane potential. That this reduction of resting membrane potential is due to the GCase deficiency in the GD2 cell lines is supported by similar results obtained from control iPSC-derived neurons in which GCase activity was inhibited by exposure to CBE and reproducible results from GD2 neurons of three patients carrying different mutations. The results show consistency with different patient lines and chemical induced GD neurons, and thus strongly support the conclusion that the phenotype reflects GD pathogenesis, and not cell line variations. It is reasonable to speculate that less negative resting membrane potential in GD2 neurons will require more pronounced depolarization for a growing steady-state inactivation of voltage-gated sodium channels which may lead to decreased neuron activity in GD.

Resting membrane potential in neurons is mainly regulated by potassium channels. The decreased potassium currents in some of GD2 cells suggest the alteration of those channel activities may contribute to the less negative resting membrane potential. This assumption is supported by transcriptome profiling of the brains from neuronopathic GD mouse model. The mRNA expression of potassium channels were decreased in the brain of these mice (unpublished data). In addition to ion channels, the function of Na+/K+-ATPase is essential to maintain the resting membrane potential. In the CNS neurons, about 40% of the energy is consumed by Na+/K+-ATPase. The energy is mainly supplied by mitochondria. Mitochondrial dysfunction has been implicated in neuronopathic GD [21,26,27]. The respiratory chain and mitochondrial membrane potential were impaired in the primary neurons and astrocytes of acute neuronopathic GD mice [26]. Inhibition of GCase by CBE leads to defective mitochondrial function in human dopaminergic cell line [27]. The mechanistic link of mitochondria function and neuronal electrophysiology properties in GD neurons warrant further investigation. In addition, ER stress and the UPR pathway have been demonstrated to affect electrophysiological activity of the motor neurons from humans with Amyotrophic Lateral Sclerosis [60]. ER stress and UPR are implicated in GD fibroblasts and Drosophila models [61–63], which could be important factors affecting neuron function in GD. Furthermore, secondary pathogenic protein aggregates (αSYN and APP) and/or substrate accumulation may cause disruptions on the membrane proteins in neurons which could affect signaling or transport in neurons. In our cultures, the neurons generated were a mix of TH-positive dopaminergic neurons and other neuron types that were not determined in this study. As a result, the abnormal electrophysiological changes likely reflect general neuronal phenotypes in GD neurons rather than DA neuron-specific effects. Taken together, this study reveals for the first time defective electrophysiological properties in GD neurons that could underlie neuronopathic GD phenotype.

Here, GD2-derived fibroblasts, iPSCs, NPCs and neurons, had major increases in glucosylsphingosine, but lesser increases of glucosylceramide. Several mechanisms could provide explanations, including alternative paths for the metabolism/production of these two substrates [58]. Unlike glucosylsphingosine, glucosylceramide levels in GD2 iPSC-derived NPC and neurons are not consistently elevated in each mutant. The proliferation rate of the cells and confluence of the cells in the dish could affect cellular glucosylceramide level. Thus, glucosylsphingosine may be a more sensitive and reliable marker for determining substrate accumulation in these cells. In GD, the specific accumulation of glucosylceramide species depends upon the *Gba1/GBA1* mutation-derived GCase, and the particular tissues and the developmental stage [57,64,65]. The glucosylceramide species distribution was not altered by the *GBA1* mutations in the iPSCs and iPSC-derived NPCs and neurons. These GD2 cells are functionally null at the lysosomal level and so, accumulated glucosylceramide species is reflective of the cells’ normal compositions.

Glycosphingolipid analyses revealed a distinct glucosylceramide species in each iPSC-derived cell type, indicating the differentiation-stage specific control of these profiles. The abundant species in fibroblasts were GC16:0, GC24:1 and GC24:0 with nearly equal representations as percent of total glucosylceramides. In NPCs, GC16:0 and GC24:1 were major species followed by GC18:0 and GC24:0. The derived neurons contained predominantly GC18:0 that is typical for brain glucosylceramides in humans and mice [57,64]. The iPSCs contained GC16:0 > GC24:1 and GC18:0 > GC20:0, which was more similar to the profile of longer acyl chains of monohexosylceramides in mouse embryonic stem cells [66]. Ceramide species profiles shift occurs during conversion of mouse embryonic stem cells to embryoid bodies, in which C18:0- and long chain fatty acyl-CoAs are increased, whereas palmitoyl-CoA derivatives (C16:0-) are less altered [66]. This result suggests that glucosylceramide metabolism in each cell type is determined by its precursor, ceramide, and the several enzymes, fatty acyl-CoA elongases and ceramide synthases that are differentially expressed in various cell types and have different substrate specificities [67,68]. Ganglioside patterns and contents are also altered during iPSC differentiation to neurons. The levels of GM3, a major ganglioside in iPSCs, are reduced in the derived neurons [33]. The total ganglioside contents were enriched in the derived neuron compared to fibroblast and iPSCs [33]. The unique glucosylceramide profiles and gangliosides contents could be useful as biomarkers for cell type differentiation in the CNS.

In summary, the generation of iPSC-derived neural populations from GD2 patient fibroblasts can provide a variety of cell models for exploring the pathologic mechanisms of neuronopathic type GDs. The findings of this study and others have demonstrated autophagic defects, impaired calcium homeostasis and abnormal electrophysiology in GD iPSCs derived neurons that facilitate the understanding of the GD pathology in CNS [31,33]. Importantly, our findings provide the first electrophysiological characterization of GD neurons that will expedite dissecting the pathological mechanisms of neuronopathic GD. The altered electrophysiological properties of GD 2 neurons suggest that affected cells may be more susceptible to activation input. The electrophysiological properties of GD iPSC-derived neurons could represent a novel area for therapeutic target screening. Furthermore, with genetic correction, these cells may ultimately be useful for the development of patient-specific cell replacement therapies for GD and Parkinson’s disease, and other neurodegenerative diseases.

## Supporting Information

S1 Fig Sequencing to confirm the *GBA1* mutations. Sequence images with named mutations above in each cell line. Chromatograms labeled control represent the reference sequence.
(JPG)

S2 Fig Reprogramming fibroblast cells to iPSCs. Representative images of GD2 and control iPSCs. (A) Immunofluorescence staining of ES cell markers Oct4 (red) and Nanog (green) in iPSCs. (B) Representative images of teratomas. H&E staining sections of teratomas from GD2-1260 iPSCs showed tissue types arising from three primordial germ layers, endoderm, ectoderm and mesoderm. The images are 400 fold magnifications. (C) GCase expression and localization in iPSCs. GCase (green) detected by anti-human GCase antibody colocalized with lysosomal marker Lamp1 (red) in control iPSCs and human ES (hES) cells. Low level GCase was in cytoplasm of GD2-1260 iPSCs and not colocalized with Lamp1.
(JPG)

S3 Fig G-banded karyotype analysis. Representative images revealing normal karyotypes (46,XX) of GD2-1260, GD2-2627, GD2-8760 and carrier iPSC lines. All the iPSC lines used for differentiation from control [40], carrier and GD2 patients had normal karyotypes.
(JPG)

S4 Fig Directed differentiation of neural cells from iPSCs. (A) Neural stem cell marker sox2 staining. NPCs of control, GD2-1260, GD2-2627, GD2-8760 and carrier expressed sox2. (B) Neuron marker NeuN and Map2 staining. Differentiated neurons of control, GD2-1260, GD2-2627 and GD2-8760 showed positive signals for NeuN and Map2.
(JPG)

S5 Fig GCase deglycosylation. Cell lysates were treated with Endo H and N-Glycanase. GCases were detected by anti-human GCase antibody. GCase in control fibroblasts, iPSCs, NPCs and neurons (14 d) were partially resistant to Endo H indicating the presence of high mannose and complex oligosaccharides on GCase. GD2-1260 GCase levels (untreated) were lower than that in control cells, and sensitive to Endo H digestion, indicating the lack of complex high mannose oligosaccharides on the mutant GCase in these cells. N-Glycanase digestion resulted in deglycosylated GCase protein band with a molecular weight ∼55 kDa in all GD2-1260 and control cell types. 40 μg protein lysate was loaded on each lane and β-actin is the loading control.
(JPG)

S1 Table Additional supporting information. See references [69–72].
(PDF)
