# Supplementary figures and images for: Properties of Neurons Derived from Induced Pluripotent Stem Cells of Gaucher Disease Type 2 Patient Fibroblasts: Potential Role in Neuropathology
Source: PLoS One. 2015 Mar 30;10(3):e0118771. doi: 10.1371/journal.pone.0118771 (PMC4378893; doi:10.1371/journal.pone.0118771)

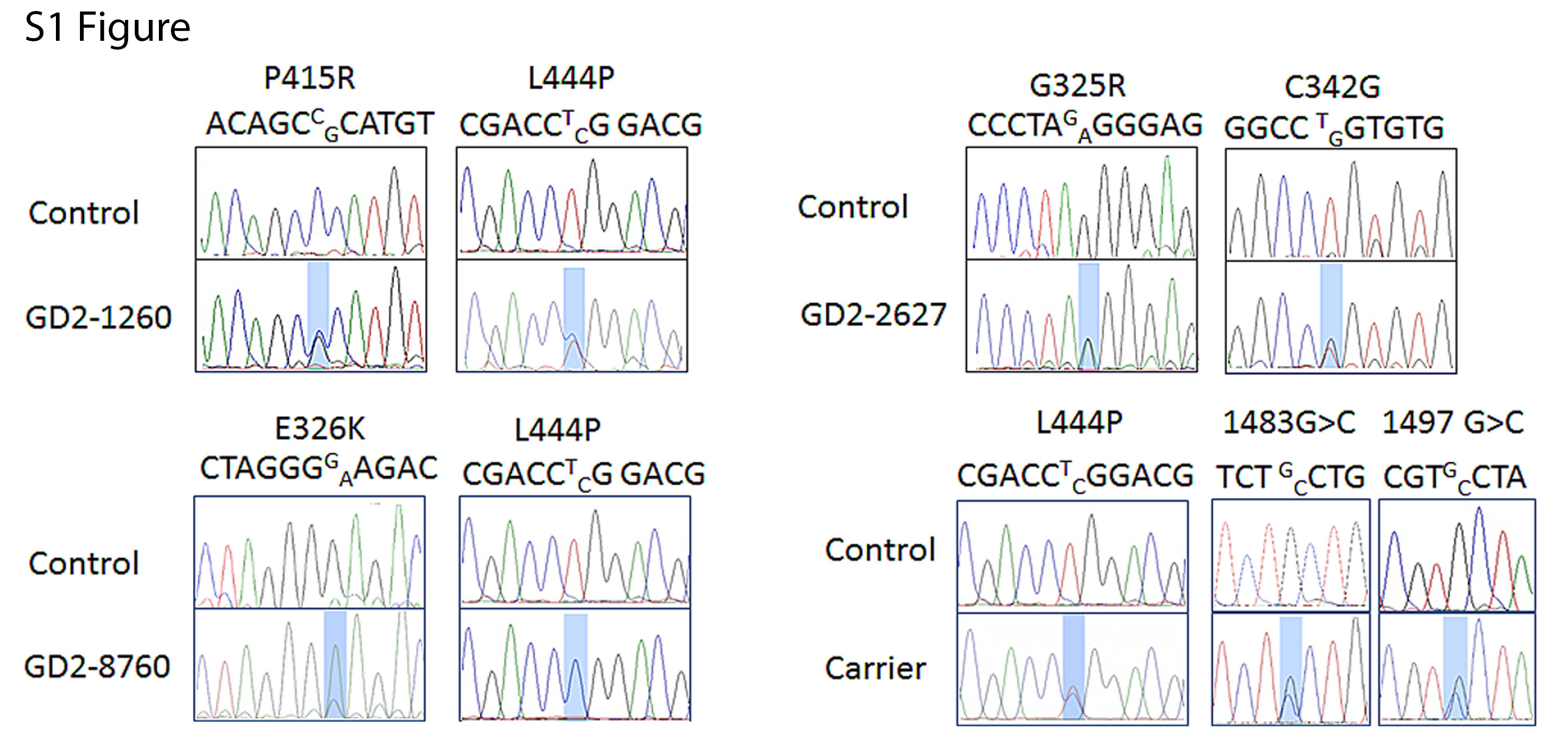

Supplement: S1 Fig — Sequence images with named mutations above in each cell line. Chromatograms labeled control represent the reference sequence. (JPG) [file pone.0118771.s001.jpg]

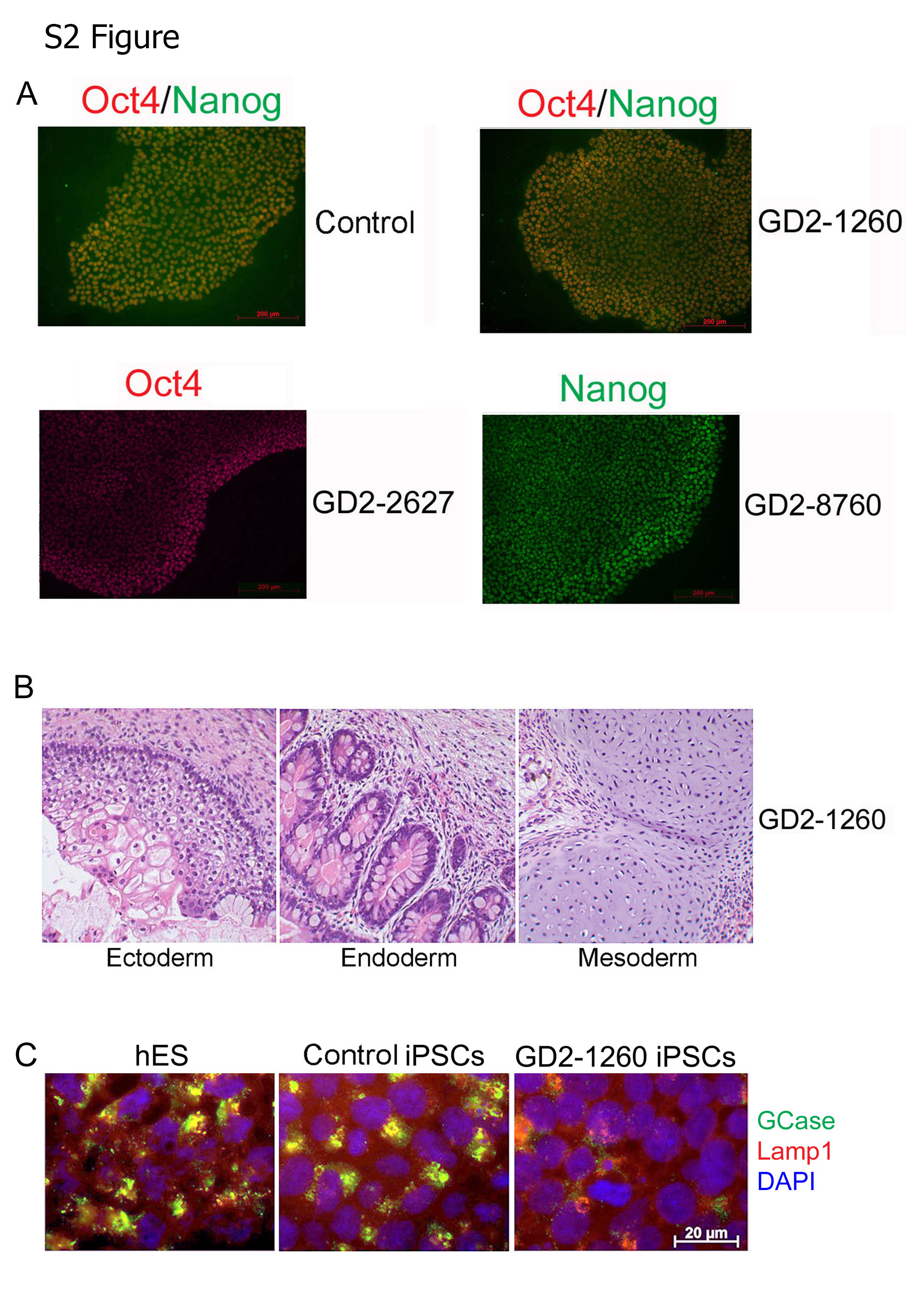

Supplement: S2 Fig — Representative images of GD2 and control iPSCs. (A) Immunofluorescence staining of ES cell markers Oct4 (red) and Nanog (green) in iPSCs. (B) Representative images of teratomas. H&E staining sections of teratomas from GD2-1260 iPSCs showed tissue types arising from three primordial germ layers, endoderm, ectoderm and mesoderm. The images are 400 fold magnifications. (C) GCase expression and localization in iPSCs. GCase (green) detected by anti-human GCase antibody colocalized with lysosomal marker Lamp1 (red) in control iPSCs and human ES (hES) cells. Low level GCase was in cytoplasm of GD2-1260 iPSCs and not colocalized with Lamp1. (JPG) [file pone.0118771.s002.jpg]

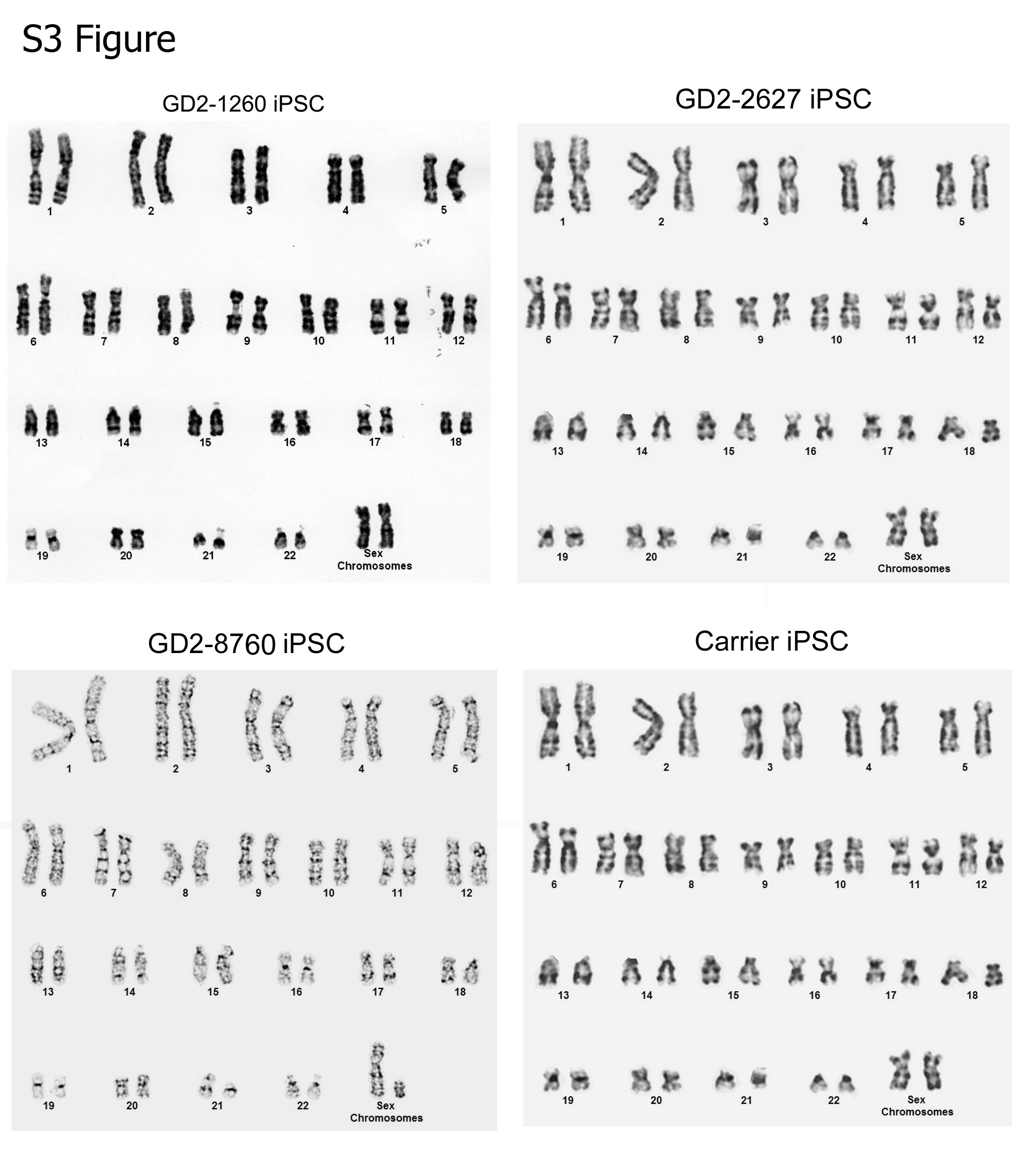

Supplement: S3 Fig — Representative images revealing normal karyotypes (46,XX) of GD2-1260, GD2-2627, GD2-8760 and carrier iPSC lines. All the iPSC lines used for differentiation from control [40], carrier and GD2 patients had normal karyotypes. (JPG) [file pone.0118771.s003.jpg]

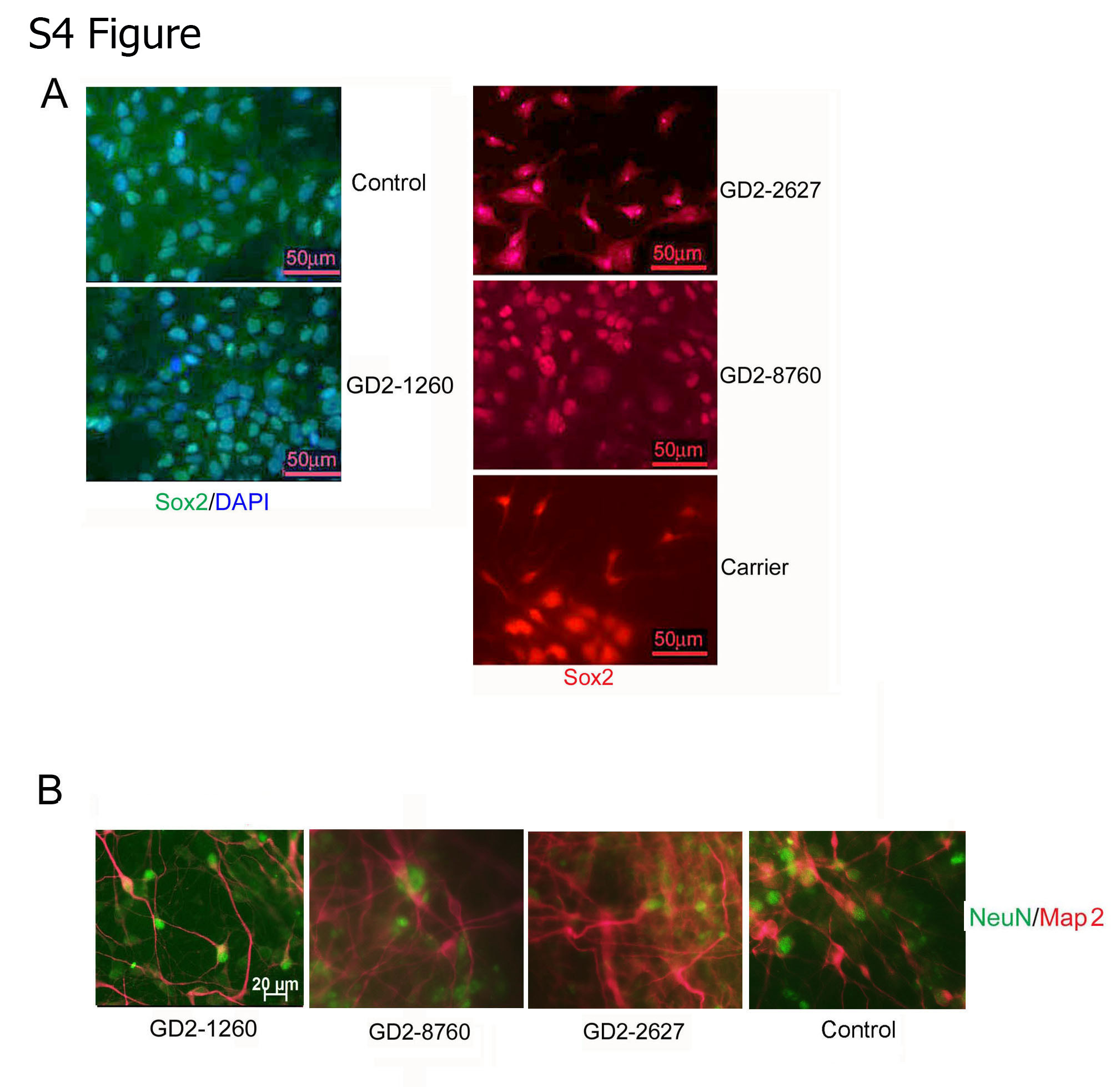

Supplement: S4 Fig — (A) Neural stem cell marker sox2 staining. NPCs of control, GD2-1260, GD2-2627, GD2-8760 and carrier expressed sox2. (B) Neuron marker NeuN and Map2 staining. Differentiated neurons of control, GD2-1260, GD2-2627 and GD2-8760 showed positive signals for NeuN and Map2. (JPG) [file pone.0118771.s004.jpg]

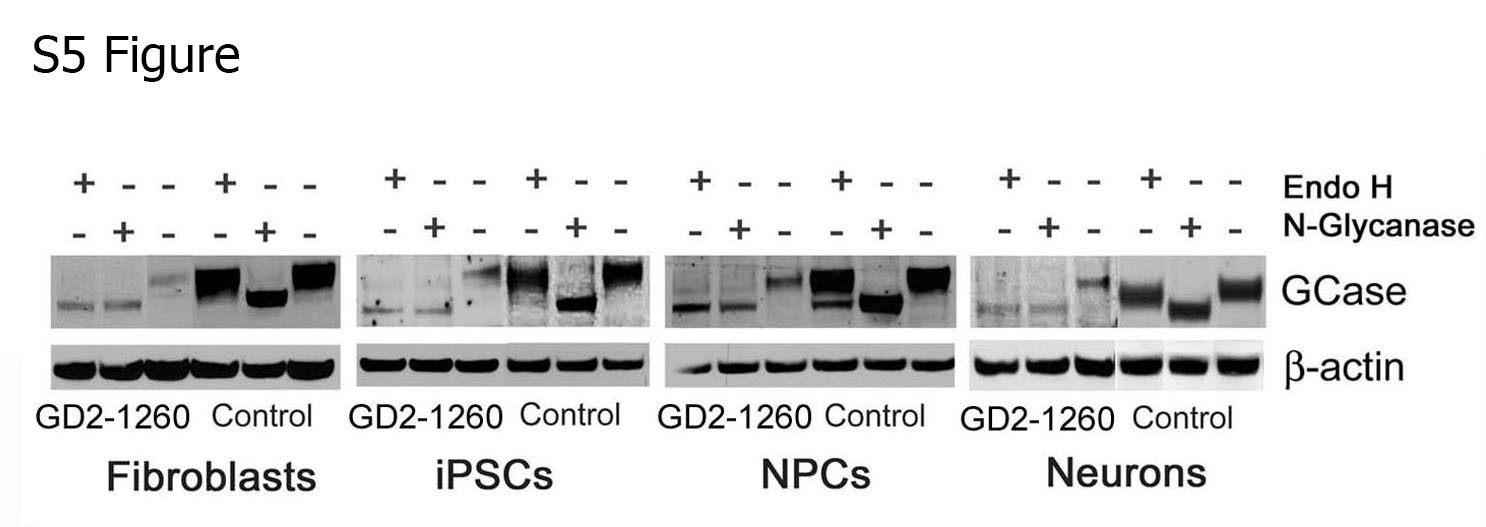

Supplement: S5 Fig — Cell lysates were treated with Endo H and N-Glycanase. GCases were detected by anti-human GCase antibody. GCase in control fibroblasts, iPSCs, NPCs and neurons (14 d) were partially resistant to Endo H indicating the presence of high mannose and complex oligosaccharides on GCase. GD2-1260 GCase levels (untreated) were lower than that in control cells, and sensitive to Endo H digestion, indicating the lack of complex high mannose oligosaccharides on the mutant GCase in these cells. N-Glycanase digestion resulted in deglycosylated GCase protein band with a molecular weight ∼55 kDa in all GD2-1260 and control cell types. 40 μg protein lysate was loaded on each lane and β-actin is the loading control. (JPG) [file pone.0118771.s005.jpg]
