# Supplementary material for: Properties of Neurons Derived from Induced Pluripotent Stem Cells of Gaucher Disease Type 2 Patient Fibroblasts: Potential Role in Neuropathology
Source: PLoS One. 2015 Mar 30;10(3):e0118771. doi: 10.1371/journal.pone.0118771 (PMC4378893; doi:10.1371/journal.pone.0118771)
Supplement: S1 Table — See references [69–72]. (PDF) [file pone.0118771.s006.pdf]

S1 Table

Table S1. Fibroblast lines from Gaucher disease patient and control human used for iPSC reprogramming										
**ID**	**Cell source**	**Diagnosis**	**Genotype**	**Race**	**Age At Sampling**	**Sex**	**Clinical notes**	**iPSC ID (# of clones)**	**Reprogramming method**	**References**
GM01260	Coriell	TYPE 2	L444P/P415R	Caucasian	11 months	Female	Splenomegaly; opisthotonus: expired at age 1; 3% of control fibroblast GCase activity.	iPSC47 (12)	Lentivirus [42]	[34,35,37-39,51]
GM02627	Coriell	TYPE 2	G325R/C342G	Caucasian	3 years	Female	Subacute neuropathic Gaucher; myoclonus; seizures; expired at age 3 due to pneumonia; 24% of control fibroblast GCase activity.	iPSC122 (6)	Episomal [43]	[34,35,37,69]
GM08760	Coriell	TYPE 2	L444P:E326K / L444P:E326K *	Caucasian	1 years	Male	Hepatosplenomegaly, retroflexion of head, strabismus, dysphagia, and hypertonicity; WBC show defect in GCase activity.	iPSC123 (6)	Episomal [43]	[38]
GM00877	Coriell	TYPE 2	L444P/L444P, 1483G>C and 1497G>C on one allele	Caucasian	1 years	Male	Hepatosplenomegaly; strabismus; trismus; expired at age 1; 4% of control fibroblast GCase activity.	iPSC126 (6)	Episomal [43]	[34-39]
GM20270	Coriell	TYPE 2	L444P/IVS2+1G>A	Other	4 months	Female	Clinically affected.	Failed to reprogram	Episomal [43]	[38,70]
GM00878	Coriell	Heterozygote	carrier for L444P/L444P, 1483G>C and 1497G>C on one allele	Caucasian	Unavailable	Female	Carrier level of fibroblast GCase activity; clinically unaffected mother of GM00877.	iPSC127 (6)	Episomal [43]	[36,71]

GM20272	Coriell	TYPE 2I	L444P/L444P		Unavailable	Male	Clinically affected; hepatosplenomegaly; slowed horizontal saccades.	Fibroblasts did not grow well	not attempted	[38]
GM01607	Coriell	TYPE 1	N370S/V394L	Caucasian	30 years	Male	Eastern European Jewish; hepatosplenomegaly since age 17; typical Gaucher's cells observed in bone marrow aspirant; 18% of control fibroblast GCase activity.	iPSC67 (4)	Lentivirus [42]	[39]
GM00852	Coriell	TYPE 1	N370S/84GG	Caucasian	20 years	Male	Adult; 6% of control fibroblast GCase activity.	Failed to reprogram	Lentivirus [42]	[39,72]
HFF10	CCHMC /PSCF	Normal human		n/a	Newborn	Male	Clinically unaffected newborn	iPSC54 (3)	Lentivirus [42]	[40]

*, Determined in this study.
